# Immediate Implant Placement and Loading in the Esthetic Zone Using a Four-Dimensional Virtual Patient Protocol: A Technical Report

**DOI:** 10.7759/cureus.108681

**Published:** 2026-05-11

**Authors:** Efstathia Afrati, Nikolaos N Giannakopoulos, Andreas Krokidis, Panagiotis Lampropoulos

**Affiliations:** 1 Prosthodontics, National and Kapodistrian University of Athens, Athens, GRC; 2 Prosthodontics, Julius-Maximilians-Universität Würzburg, Würzburg, DEU; 3 Endodontics, National and Kapodistrian University of Athens, Athens, GRC

**Keywords:** digital workflow, dynamic occlusion, esthetic zone, immediate implant placement, mandibular motion tracking, provisionalization, virtual patient

## Abstract

Rehabilitation of the anterior region requires careful prosthetic planning in cases of immediate implant placement and loading due to the complexity of prosthetically driven restoration in the esthetic zone. The management of interferences during mandibular excursive movements remains critical. A technique is presented for obtaining four-dimensional (4D) virtual patient data, where the 4D component refers to the integration of real-time mandibular movements using an optical jaw motion tracking system. Cone beam computed tomography records, intraoral and face scanning and dynamic jaw motion tracking data, including border movements, which are obtained from JMA Optic System, are superimposed using Exocad software, while data alignment is achieved using the remaining dentition as reference structures. This report describes a step-by-step method to transfer the digital treatment planning simulation into the patient’s mouth using a computer-aided design/computer-aided manufacturing (CAD/CAM)-fabricated surgical guide for implant placement and a digitally designed provisional restoration to facilitate clinical reproduction of the planned prosthetic position, supporting a prosthetically driven approach to anterior implant rehabilitation.

## Introduction

Smile design within the prosthodontic plan for the maxillary anterior area has mainly relied on conventional planning approaches that combine clinical assessment with analog records for the evaluation of esthetic and functional outcomes. However, these conventional approaches may not completely integrate all anatomical, esthetic and functional parameters within a comprehensive treatment plan, which appears particularly challenging in implant placement cases, when an esthetically favorable emergence profile of the soft tissues has to be achieved [1-5]. To establish long-term success of the implant-supported rehabilitation, precise patient-specific planning is required [6], particularly regarding alveolar ridge morphology, gingival biotype, facial esthetics, as well as mandibular kinematics. Fully digitally developed workflows have been increasingly incorporated into implant dentistry to enhance the precision and predictability of surgical procedures while providing simulation of the treatment outcome and the potential of adaptation to the patients' expectations. However, it should be acknowledged that these workflows are susceptible to registration errors and device-related accuracy limitations that may affect the subsequent prosthetic outcome [7,8].

In recent years, the concept of three-dimensional (3D) virtual patient models has been well reported for the rehabilitation of the esthetic zone [9-11]. By incorporating superimposed data from intraoral scanners (IOS), facial scanning (FS) and cone-beam-computed tomography (CBCT), the visualization of prosthetic rehabilitation can be enhanced [12-14]. Nevertheless, current digital workflows are predominantly based on static records, which may not inherently address interferences during mandibular excursions, whereas emerging approaches increasingly incorporate functional data, such as mandibular motion analysis [15]. The integration of a fourth dimension (4D) - defined as time-dependent mandibular motion data - into digital planning has been proposed as a means to potentially improve esthetics and functional rehabilitation and can be achieved by importing functional data such as mandibular movement registrations in the design software to facilitate functional contact simulation [16-19]. Recording the dynamic movements of the mandible by using a jaw-tracking system provides useful information for the digital protocol, particularly regarding the capture of individualized patient mandible kinematics to efficiently adjust the virtual design of definite restoration. The 4D workflow may present enhanced patient convenience while potentially allowing a more detailed assessment of functional contacts compared to conventional static records [15,20], provided that accuracy is supported by precise calibration and high registration quality of the specific jaw motion tracking system used.

This technical report presents a step-by-step complete digital workflow for immediate implant placement, provisionalization and immediate non-occlusal loading in the esthetic zone, specifically in the anterior maxilla region. This approach incorporates dynamic mandibular motion data into the virtual planning process while combining conventional digital records, such as intraoral and face scans and CBCT data to create a 4D virtual patient (4DVP). The presented workflow follows a prosthetically guided concept, in which implant placement and provisional restoration design are based on esthetic and functional parameters. Considering the clinical challenges of immediate implant placement in esthetically demanding areas, this technique aims to reduce chairside occlusal refinements and enhance emergence profile contouring. Nevertheless, clinical verification remains essential to validate intraoral occlusal contacts as well as the proper facial aesthetics assessment.

This article was previously presented as an e-poster at the 2026 Annual Conference of the Greek Prosthodontic Society on February 14, 2026.

## Technical report

**Figure 1:**
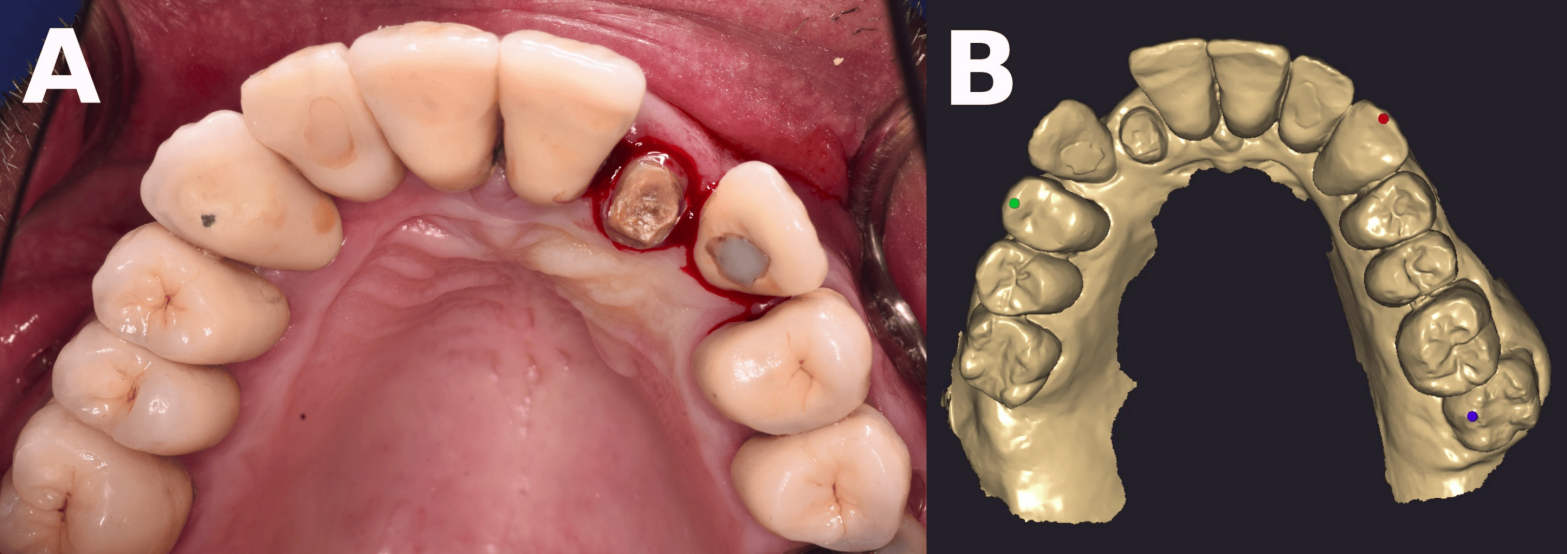
A. Preoperative intraoral (occlusal view of maxilla); B. Intraoral scanning data

**Figure 2:**
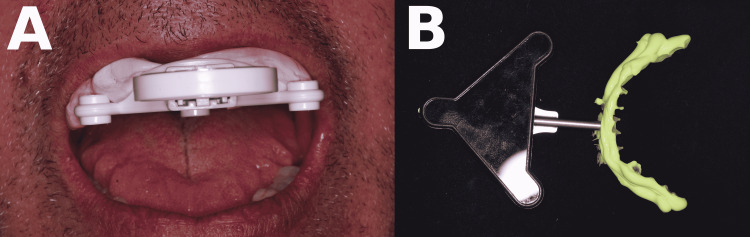
A. Putty impression material adapted to the bite fork for dynamic occlusion recording; B. Mandibular motion registration using bite impression material. Secure fixation of the para-occlusal attachment appears essential for the efficient transfer of mandible trajectories to the software.

**Figure 3:**
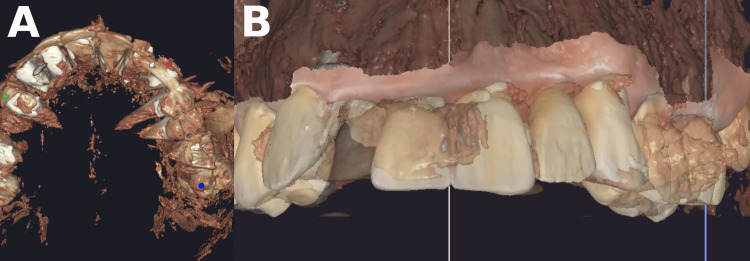
A. CBCT integration into the software program and selection of anatomical anterior and posterior reference markers; B. Superimposition of CBCT-IOS files (frontal view). Proper alignment of incisal edges enhances the registration accuracy CBCT: Cone Beam Computed Tomography, IOS: Intraoral Scanning

**Figure 4:**
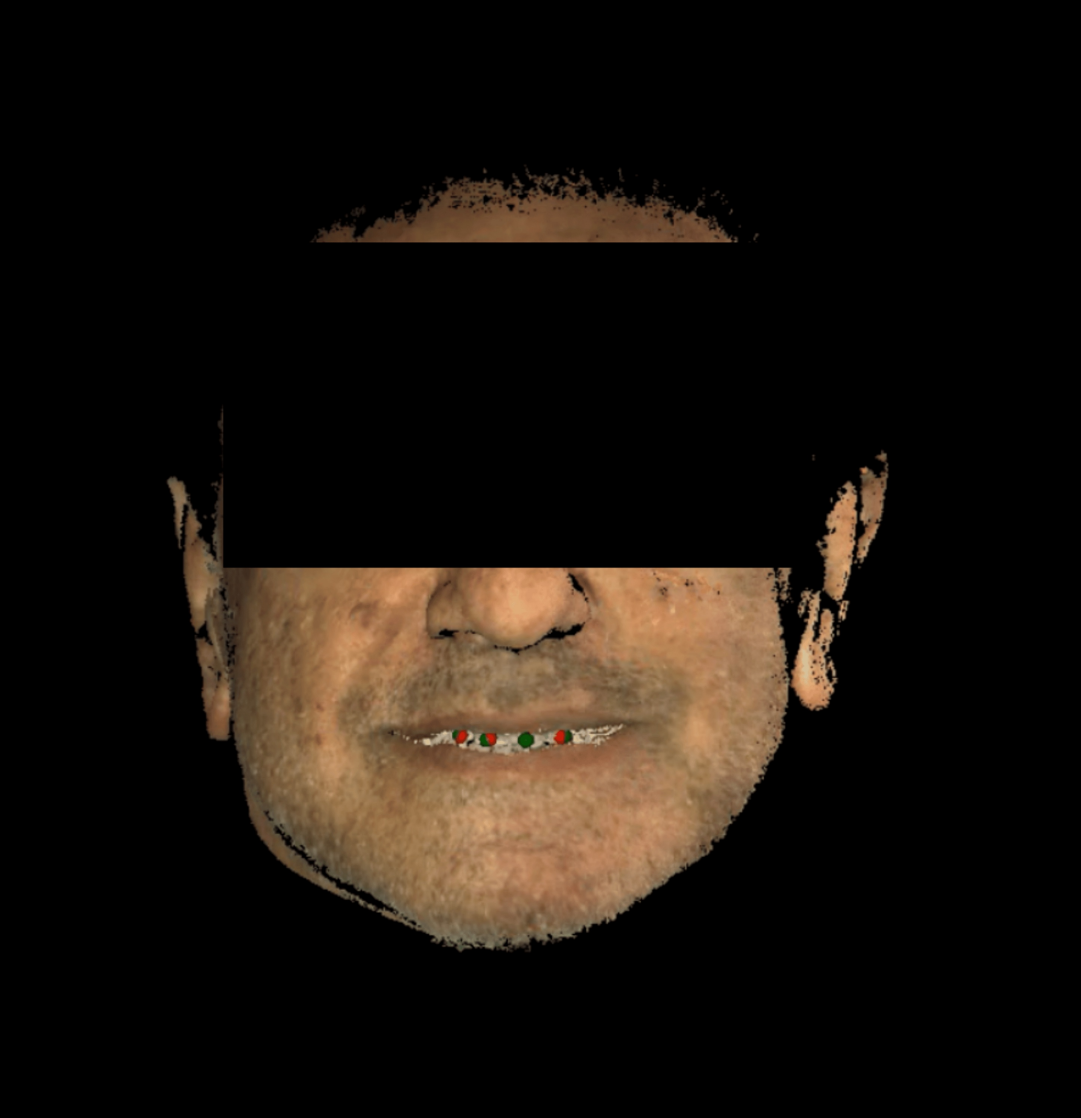
Integration of face scanning data. The maxillary anterior teeth are used as reference landmarks for the alignment with the CBCT and IOS data CBCT: Cone Beam Computed Tomography, IOS: Intraoral Scanning

**Figure 5:**
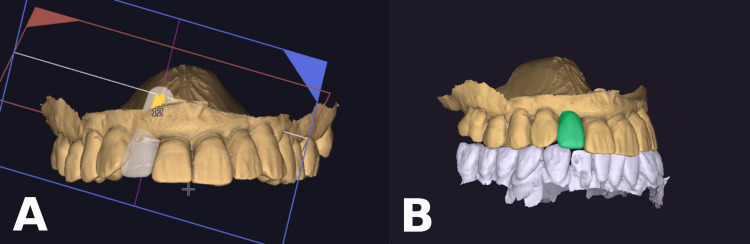
A. Virtual implant position planning relative to the alveolar ridge anatomy and the overall four-dimension (4D) virtual patient data; B. Provisional restoration design

**Figure 6:**
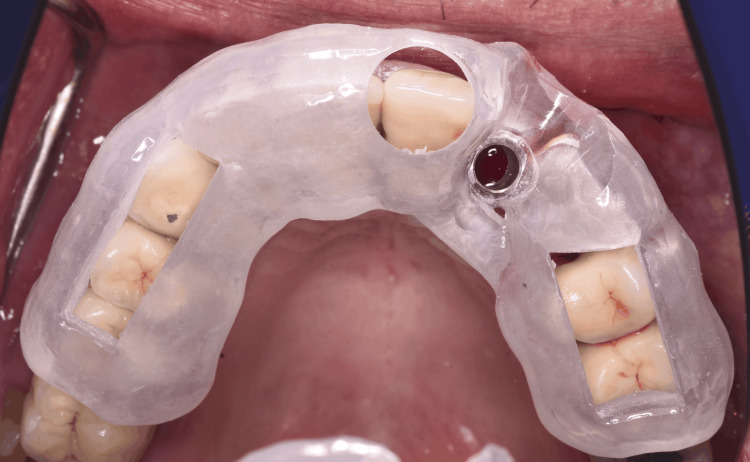
Immediate atraumatic flapless extraction of the root fragment and guided implant placement through the surgical template

To illustrate the technique, the case of a 65-year-old male patient, classified as American Society of Anesthesiologists (ASA) II with a stable, habitual occlusion, presenting with a maxillary right lateral incisor fracture is reported. Before treatment planning, extraoral, intraoral and radiographic examination was conducted. A horizontal root fracture, located at the cervical third, was identified through clinical and periapical radiographic examination. The presence of a detached coronal fragment further confirmed the diagnosis. Clinical examination revealed probing depths within normal limits and signs of slightly increased mobility, classified as Grade I. However, the remaining tooth structure was insufficient to provide at least 2 mm of circumferential ferrule, as the fracture margin was located at the equigingival level with minimal supragingival sound tooth structure, leading to a compromised prognosis. A conservative restorative approach using post-and-core and full coverage restoration was considered unfavorable due to an inadequate crown-to-root ratio. Considering all the above, tooth extraction, immediate guided implant placement, provisionalization and immediate non-occlusal implant loading were determined. Written consent was obtained from the patient for the use of clinical information and associated images in a scientific context. The technique is presented in the following steps:

1. Obtain intraoral and extraoral photographs of the initial situation in frontal, lateral and occlusal view using a digital single-lens reflex camera (D7200; Nikon Corp., Tokyo, Japan) equipped with a ring flash system, (Nissin MF18; Nissin Digital, Tokyo, Japan) and a cheek retractor (Columbia Cheek Retractor; Hu-Friedy Group, Chicago, IL, USA) (Figure 1). Perform CBCT (CS 8100 3D; Carestream Dental, Atlanta, GA, USA) of the maxilla using 8x5 cm field of view (FOV). Use acquisition parameters of 150 μm voxel size, 90 kV, 3.2 mA and 15 seconds exposure time. Position and stabilize the patient according to the manufacturer’s standard protocol.

2. Scan of both dental arches and bite registration in maximal intercuspal position (MIP), using an intraoral scanner (Medit i700, Medit Corp., Seoul, South Korea). Acquire a single-bite scan and check intercuspation contacts intraorally to verify occlusal accuracy. Export the intraoral scans (IOS) as standard tessellation language (.STL) files to capture high-resolution geometry (Figure 1).

3. Collect face scan (FS) data with a portable face scanner (Thunk 3D, Beijing Xunheng Technology Co., Ltd., Beijing, China). Scan the entire patient’s face with the patient in an upright position and the head facing forward at a 40 cm distance under constant, ambient indoor lighting. Capture separate scans of rest and posed smile and instruct the patient to remain still to avoid motion artifacts and possible alignment errors. Evaluate the smile line based on the posed smile and use this frame as the reference for esthetic planning. Export the 3D face images as OBJ files.

4. Acquire functional mandibular movements using an optical jaw motion analyzer (JMA Optic; Zebris Medical GmbH, Isny im Allgäu, Germany). Position the dedicated bite fork with a minimal amount of putty silicone impression material (Silagum Putty Soft, DMG, Hamburg, Germany) sufficient to achieve stable adaptation, on the occlusal surface of the full arch maxillary teeth. Trim any excessive silicone material from the buccal and labial surfaces. Scan intra- and extraorally and stabilize the para-occlusal attachment on the labial surfaces of the mandibular teeth using bite registration material (Luxabite, DMG, Deutschland) and verify stability clinically before recording by the absence of displacement during mandibular elevation (Figure 2). Record the border jaw movements, including opening, closing, protrusion and lateral excursions, each performed at three repetitions. Import them in extensible markup language (.XML) format into Exocad software (Exocad DentalCAD 3.1 Rijeka, developed by exocad GmbH, Darmstadt, Germany). Use the Virtual Articulator module to simulate the dynamic excursive movements and refine occlusal contacts and incisal guidance. Cross-verify the occlusion pathway intraorally using 21 μm articulating paper (AccuFilm II, Parkell, Inc., Edgewood, NY, USA) to ensure alignment.

5. Import the Dicom images (.dcm) from the CBCT into the CAD design software and superimpose with the .stl files from the IOS (2D). Perform data registration in Exocad using surface-based best-fit alignment of soft and hard tissue structures. Verify alignment by visual assessment using defined reference checkpoints in the anterior and posterior areas (Figure 3).

6. Superimpose the CBCT, IOS and OBJ files to create a three-dimensional virtual patient (3DVP). Use the maxillary anterior teeth as reference markers and verify the incisal edges overlap to achieve alignment (Figure 4).

7. Combine IOS, FS (EOS), CBCT dicom converted to STL after threshold-based segmentation with manual refinement in the Εxoplan software (Exoplan; exocad GmbH, Darmstadt, Germany). Import the patient’s specific jaw motion trajectories in order to manage dynamic occlusion and adjust the restoration’s contact design and incisal guidance to complete the creation of the 4DVP.

8. Conduct virtual implant planning by selecting the dedicated digital library (MIS Implant Technologies, Bar-Lev Industrial Park, Israel) using Exoplan software. Select the implant system (C1 Implant System, MIS Implant Technologies) and incorporate the subsequent temporary cylinder geometry from the library concurrently with implant positioning (Figure 5). Based on that, design a full arch tooth-supported surgical guide in the CAD software with occlusal inspection windows and a metallic sleeve according to the implant’s placement surgical protocol. 3D-print the guide using a methacrylate-based 3D printing resin (Keyguide, Keystone Industries, Gibbstown, NJ, USA) followed by a two-stage isopropanol bath process. Remove the support structures, trim any residual liquid resin from the printed part and perform final light-curing polymerization, according to the manufacturer’s instructions. Verify the guide’s clinical seating visually through the windows. Design and 3D print the screw-retained composite resin provisional prosthesis (SprintRay EU Temp Crown & Teeth Resin, SprintRay Inc., Los Angeles, CA, USA) (Figure 5).

9. Extract atraumatically the upper right lateral incisor root fragment according to a flapless approach, using a kit of straight and curved periotomes (Interchangeable Periotome Kit; Hu-Friedy Group) while applying minimal pressure to preserve the buccal plate. Place the dental implant according to a fully guided surgical protocol through the template, securing primary stability using a 50 Ncm insertion torque and verify with an implant stability quotient (ISQ) value of 74 (Figure 6).

10. Place the prefabricated composite resin provisional restoration on a temporary titanium cylinder (CS, MIS Implant Technologies). Select a 3-mm gingival height for the temporary cylinder, based on the implant’s platform depth and mucosal thickness to support aesthetics and enhance the emergence profile of the peri-implant soft tissues. Follow a non-occlusal loading protocol by adjusting the restoration out of dynamic occlusion contacts.

11. Maintain the non-occlusal provisional restoration for a three-month healing period. Remove the provisional and place a scan post (CS, MIS Implant Technologies) on the implant and scan with the same intraoral scanner (Medit i700) the implant position, the opposing arch and the occlusal relationship.

12. Import the scan files data into the Exocad software. Design and fabricate the definite screw-retained restoration by milling a monolithic zirconia block (3D Pro Zir, Aidite Technology Co, Ltd, Qinhuangdao, China). Use a Ti-base (CS, MIS Implant Technologies) bonded laboratory to the prosthesis, according to the manufacturer’s guidelines. (Figure 7). Verify the passive fit through periapical radiography.

**Figure 7:**
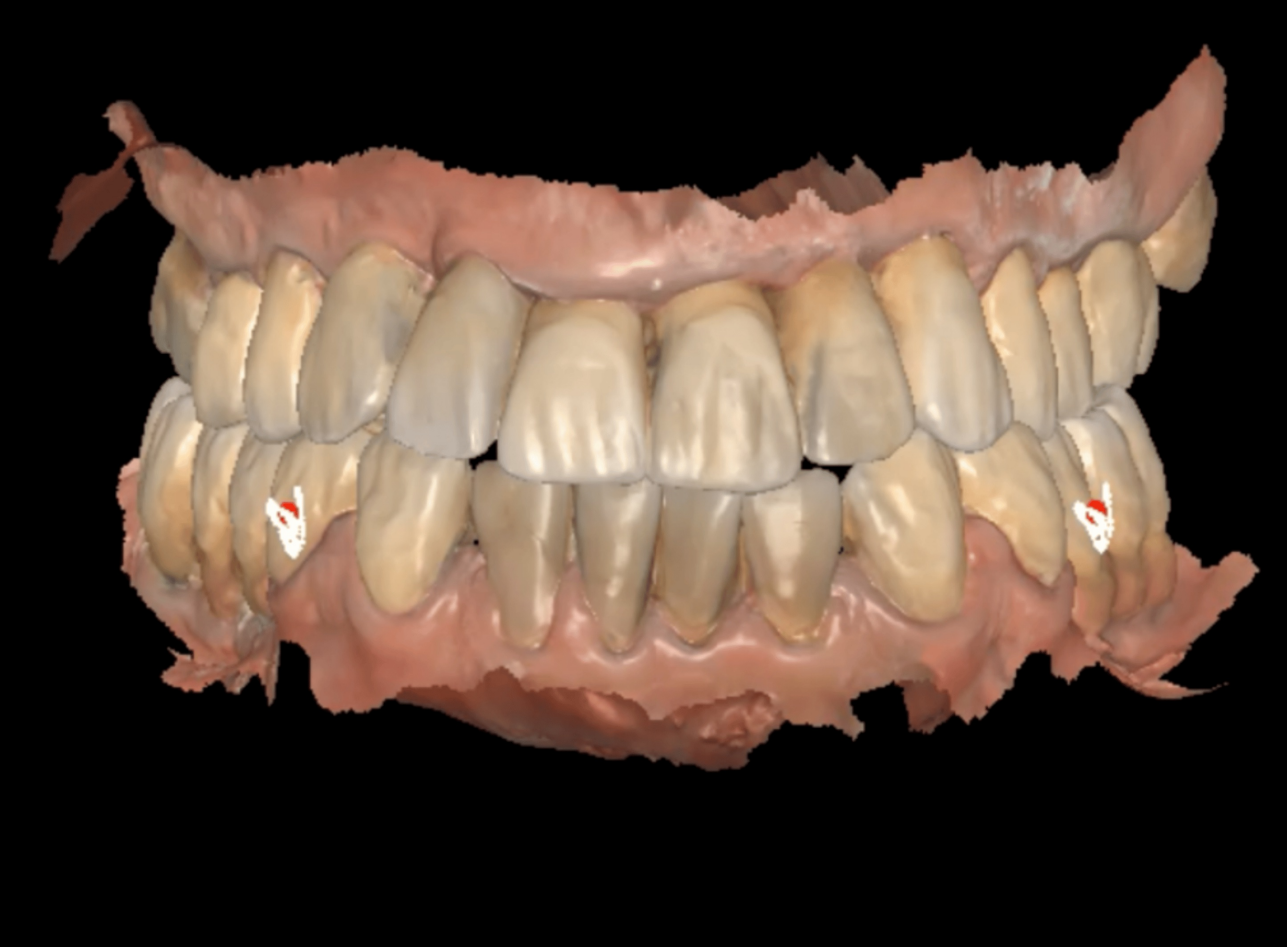
Design of the definite prosthetic restoration

**Figure 8:**
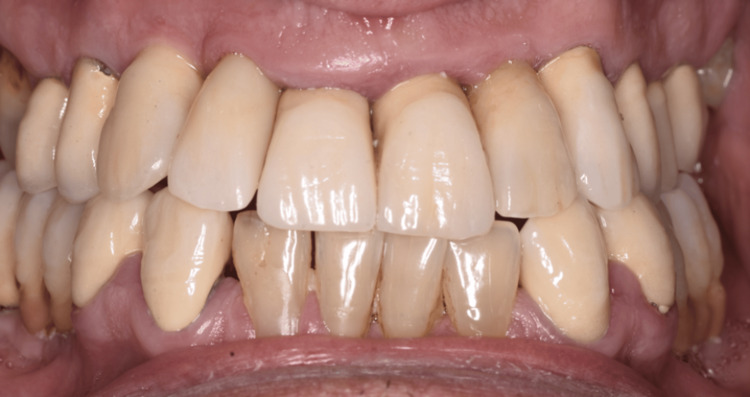
Final intraoral prosthesis of the right maxillary lateral incisor

13. Deliver the final restoration, tighten the abutment screw to 30 Ncm and seal the screw access channel with sterilized polytetrafluoroethylene (PTFE) tape and light-cured flowable composite resin (Filtek Supreme Flowable, 3M Oral Care Division, St. Paul, MN, USA). Evaluate the occlusion using 21 μm articulating paper (AccuFilm II, Parkell, New York) to identify and address potential functional interferences, following with an 8 μm occlusion foil (Shimstock; Coltene/Hanel, Altstätten, Switzerland) for a final verification of intercuspation contacts. Perform surface finishing using ultra-fine diamond burs (Komet Dental, Lemgo, Germany) and polish resin surface with a specialized polishing system (Opti1Step, Kerr Corporation, Brea, CA, USA) (Figure 8). Evaluate occlusal stability and peri-implant soft tissue integrity at six and 12 months, including periapical radiographic examination of marginal bone levels.

## Discussion

The concept of the 4D virtual patient, introduced by Lepidi et al. [16], extends the conventional 3D virtual model by incorporating dynamic mandibular motion data as the fourth dimension. While conventional workflows focus mainly on the alignment of CBCT data, intraoral and facial scanning records, the 4D approach integrates real-time kinematic data, including the capture of border movements and functional paths through optical jaw motion tracking. Based on this approach, the described technique applies these principles within a clinical protocol that integrates prosthetic and surgical parameters to achieve optimal aesthetic and functional rehabilitation. The use of 4D simulation may improve the planning precision of the subsequent implant position by incorporating individual functional movements. This workflow reduces the need of use of mechanical articulators and enables a flapless approach, with the clinical verification demonstrated by the passive fit of the surgical guide and the final prosthesis, as well as the minimal need for occlusal adjustments during functional movements after the final restoration delivery. Conventional prosthetic workflows relying on mechanical articulators may introduce inaccuracies in occlusal reproduction [15] and are difficult to integrate within a fully digital approach. On the contrary, the use of jaw motion data allows the design of a more precise tooth morphology, particularly in the palatal surfaces [15], in this case by defining the palatal guidance surfaces based on the patient’s functional incisal contact pathways. This allowed the integration of dynamic excursions into the design, which was clinically evaluated using articulating paper to confirm the absence of interferences after delivery. However, immediate implant placement in the anterior maxilla appears challenging and can be a technique-sensitive procedure, influenced by anatomical factors such as labial bone thickness, alveolar bone concavity and width of the remaining keratinized mucosa [1,4]. In the present technique, the patient exhibited a thick gingival phenotype with a buccal plate thickness of 2 mm and adequate keratinized tissues, factors that favored the decision for an immediate implant placement approach. Therefore, comprehensive treatment planning and three-dimensional radiographic evaluation is considered essential.

Among the advantages of this reported technique are included the potential for reduced intraoperative challenges and a more predictable aesthetic and functional outcome, achieved through enhanced dentist-dental technician-patient communication. Incorporating mandibular kinematics enables the integration of patient-specific parameters such as anterior guidance and condylar movement [15,17,19]. This approach offers a potential application for complex cases such as bruxism to manage dynamic occlusion [20]. Nevertheless, the effect on masticatory forces remains to be validated through further comparative studies.

However, the limitations of this method are mainly associated with the cost of advanced software and hardware equipment to process patient data, while increased laboratory time might be required. Inaccuracies related to intraoral scanning procedure have also been reported [7], particularly regarding the bite and inter-arch relation registration during scanning. To mitigate such factors, a standardized scanning strategy according to the manufacturer’s guidelines was performed in this technique, supplemented by visual inspection of the intercuspation contacts intraorally to confirm accuracy. Furthermore, the existing jaw tracking systems may present limitations related to accuracy, equipment requirements, and recording conditions [17]. These discrepancies, which may accumulate during the different workflow stages, may influence the final rehabilitation outcome. To manage these discrepancies in this technique, quality control was performed by verifying the CBCT-IOS alignment and cross-referencing the imported motion data with the patient’s clinical occlusal contacts.

The reported method may be particularly indicated in anterior immediate implant cases where immediate implant placement, provisionalization, and patient-specific functional morphology are required. However, the technique’s predictability may be challenged in regions where soft-tissue quality is compromised and may potentially require complicated tissue-augmentation procedures. Furthermore, mandibular motion tracking may appear technically demanding in cases of deep bite malocclusion, as the increased vertical overlap may limit the available space for the placement of tracking components or compromise the visibility of alignment reference markers. Therefore, careful case selection is considered critical.

## Conclusions

This article presents a workflow for immediate implant placement, provisionalization and immediate non-occlusal loading in the anterior esthetic zone that incorporates the generation of a 4D digital patient, while allowing the transfer of the digital treatment planning to the clinical setting. The technique allows the integration of patient-specific data into the virtual design process. Therefore, the procedure may facilitate prosthetically guided implant placement in selected esthetically demanding anterior regions and in cases requiring immediate treatment. However, as this workflow is demonstrated through a single case without comparative data, further clinical studies evaluating accuracy, efficacy and predictability are required before broader clinical adoption.
